# Double coronary stent loss with aortic migration and iliac embolization successfully managed by snare retrieval: a case report

**DOI:** 10.3389/fcvm.2026.1909146

**Published:** 2026-08-06

**Authors:** Muhammad Saad Reihan, Ahmed Mohammed Ahmed Mostafa, Tarek Ahmed Ahmed Dabash, Waseem Omar Ahmed, Husna Irfan Thalib, Mohammed Ali Mohammed Hammad, Hamdy Abdelazeem Elkafory, Mohammed Moanes Mohammed Mohyeldin

**Affiliations:** 1 Cardiology Department, Faculty of Medicine, Al-Azhar University, New Damietta, Egypt; 2 Department of Internal Medicine, General Medicine Practice Program, Batterjee Medical College, Jeddah, Saudi Arabia; 3 Hayat Hospital, Riyadh, Saudi Arabia; 4 General Medicine Practice Program, Batterjee Medical College, Jeddah, Saudi Arabia; 5 Cardiology Department, Faculty of Medicine, Kafr Elsheikh University, Kafr El-Sheikh, Egypt; 6 Cardiology Department, Faculty of Medicine, Al-Azhar University, Cairo, Egypt; 7 Hayat Hospital, Jazan, Saudi Arabia

**Keywords:** coronary embolization, LAD, PCI complication, snare retrieval, stent loss

## Abstract

**Background:**

Coronary stent loss during percutaneous coronary intervention (PCI) is rare but potentially life-threatening, particularly when device deformation is accompanied by aortic migration or systemic embolization.

**Case presentation:**

A 45-year-old man with diabetes mellitus and hypertension presented with high-risk unstable angina. Angiography demonstrated a critical calcific proximal left anterior descending (LAD) lesion followed by a long distal lesion. After successful proximal LAD stent deployment, a second stent failed to cross the proximal edge of the implanted stent because of strut interaction and snagging. Repeated manipulation caused entrapment, elongation, and severe deformation of the undeployed stent, which protruded into the ascending aorta. The patient developed transient hypotension, sinus bradycardia, and vomiting and was stabilized with intravenous fluids, atropine, and norepinephrine. Snare retrieval through femoral access captured the deformed stent; however, traction dislodged the previously implanted proximal LAD stent, which embolized to the right common iliac artery bifurcation. Urgent computed tomography localized the embolized stent. During the same procedural session, briefly interrupted for imaging, both stents were successfully retrieved using an Amplatz GooseNeck snare through bilateral femoral access. Definitive LAD PCI was then completed with overlapping drug-eluting stents, restoring TIMI 3 flow without residual stenosis. The complete procedure was accomplished within approximately 3 h.

**Conclusion:**

Sequential loss of two coronary stents with aortic migration and iliac embolization is exceptionally uncommon. Careful hemodynamic stabilization, prompt cross-sectional imaging, and stepwise snare retrieval can permit successful single-session percutaneous management and avoid emergency surgery.

## Introduction

Stent loss during percutaneous coronary intervention (PCI) is an uncommon complication in contemporary practice, with a reported incidence of less than 1% in the era of low-profile, premounted stent systems (1). Although infrequent, an unexpanded or dislodged stent may produce serious consequences, including abrupt coronary occlusion, myocardial infarction, stroke, systemic embolization, and peripheral arterial obstruction (2).

Predisposing factors include severe vessel calcification, tortuosity or angulation, inadequate lesion preparation, insufficient guide-catheter support, and interaction with the struts of a previously implanted stent (3, 4). Management depends on the stent location, whether guidewire access is preserved, the risk of further migration, and the patient's hemodynamic condition. Percutaneous options include small-balloon retrieval, loop-snare capture, and crushing or deployment of the lost stent against the vessel wall; surgical removal is generally reserved for situations in which percutaneous management is unsuccessful or unsafe (2, 5).

We report a rare case of sequential loss of two coronary stents during LAD intervention. An undeployed distal stent became entrapped at the proximal edge of a previously deployed LAD stent, elongated, and migrated into the ascending aorta. During snare retrieval, the deployed proximal stent was secondarily dislodged and embolized to the right common iliac artery bifurcation. Both stents were retrieved percutaneously, and definitive LAD revascularization was completed during the same procedural session.

## Case presentation

### Ethics approval

Ethical approval for publication of this case report was obtained from the institutional ethics committee in accordance with institutional and international ethical standards.

### Informed consent

Written informed consent was obtained from the patient for publication of this case report and accompanying images.

A 45-year-old man with a long-standing history of Non-Insulin Dependent diabetes mellitus (NIDDM) and hypertension presented with severe retrosternal chest pain of 4 h' duration. The pain was progressive and agonizing in nature, prompting referral from a peripheral hospital for urgent coronary angiography with a working diagnosis of high-risk acute coronary syndrome–unstable angina. On admission, physical examination, including general and cardiovascular assessment, was unremarkable, and the patient was hemodynamically stable.

Electrocardiography demonstrated 1-mm horizontal ST-segment depression in leads V1–V6, suggestive of anterior wall ischemia. Initial cardiac biomarkers, including troponin levels, were within normal limits. Transthoracic echocardiography revealed preserved left ventricular systolic function without regional wall motion abnormalities or significant valvular disease. Prior to transfer, the patient received aspirin and intravenous morphine for pain control.

### Initial coronary intervention

**Figure 1:**
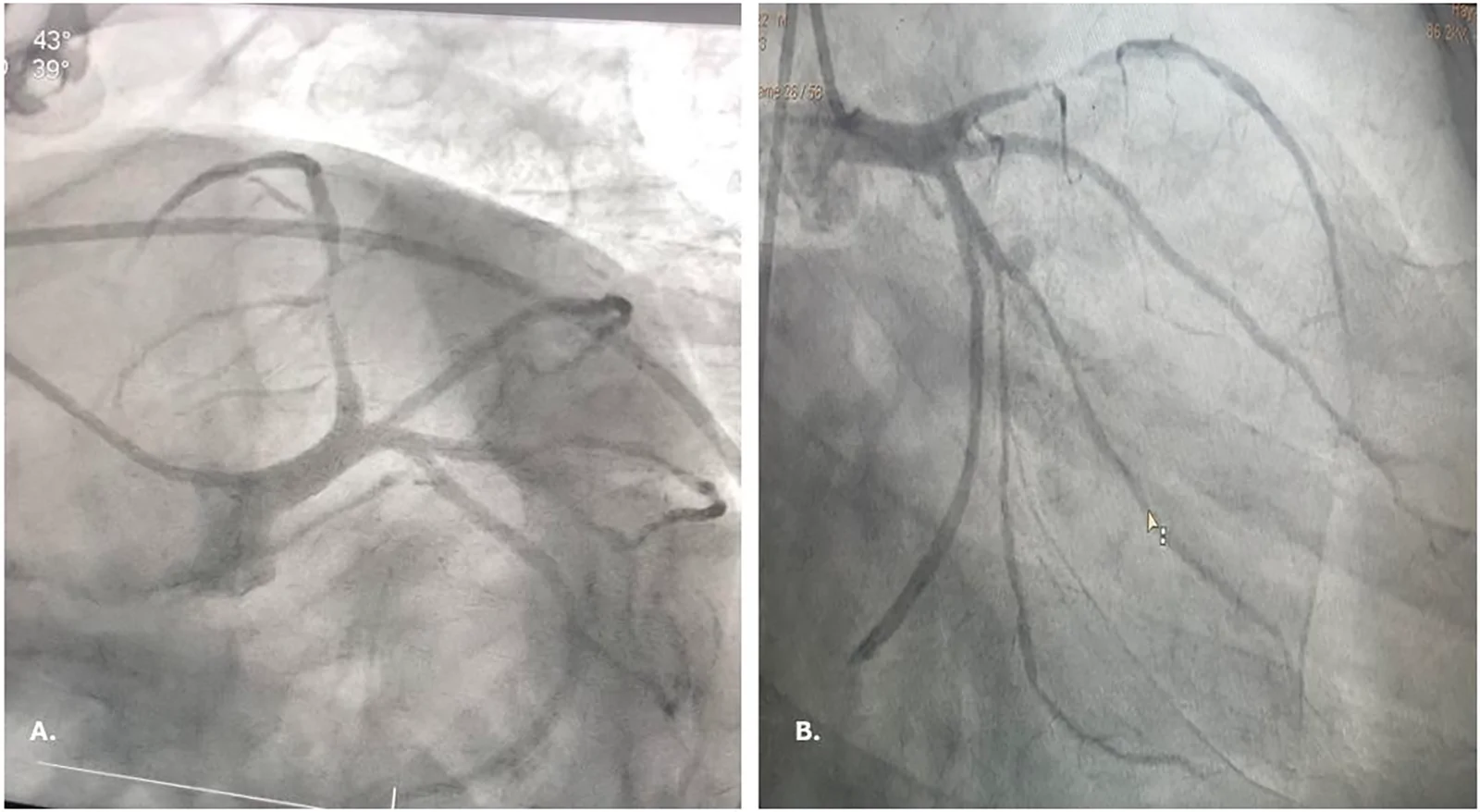
**(A)** Left anterior oblique with caudal angulation angiographic view (spider) showing critical 99% proximal LAD stenosis. **(B)** Right anterior oblique with caudal angulation angiographic view showing tight calcific proximal LAD stenosis.

Coronary angiography demonstrated a critical proximal 99% LAD lesion (Figures 1A,B). A drug-eluting stent was successfully deployed in the proximal LAD.

Urgent coronary angiography and percutaneous coronary intervention (PCI) were performed via right femoral arterial access using a 6F sheath. Coronary angiography demonstrated a right-dominant coronary circulation with a critical 99% proximal subtotal occlusion of the left anterior descending (LAD) artery followed by a long significant lesion (Figure 1). The left main coronary artery was angiographically normal, while the left circumflex artery showed mild nonobstructive disease and the right coronary artery demonstrated a borderline distal lesion planned for conservative follow-up.

**Figure 2:**
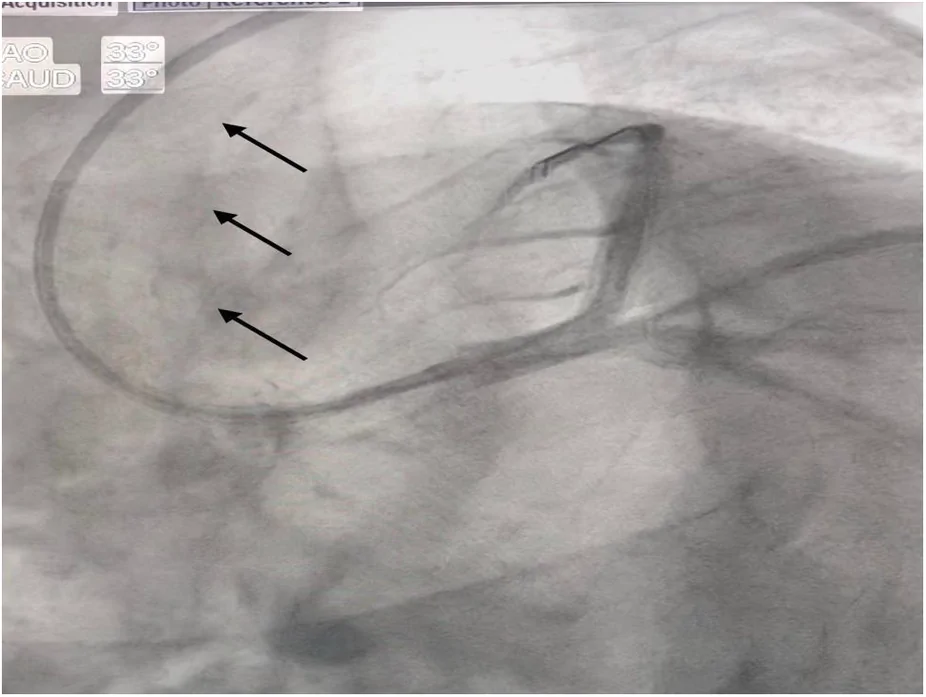
Left anterior oblique with caudal angulation angiographic view (spider) demonstrating the stretched and deformed stent protruding into ascending aorta (black arrows).

PCI to the LAD was undertaken using a 6F Launcher EBU 3.5 guiding catheter. The lesion was crossed using a PT2 MS guidewire and predilated with a 2.0 mm × 25 mm Sprinter balloon catheter (Medtronic, Minneapolis, MN, USA). followed by deployment of a 3.5 mm × 34 mm Resolute Integrity zotarolimus-eluting stent (Medtronic, Minneapolis, MN, USA). At 16 atm with satisfactory angiographic expansion. Following proximal stent implantation, an additional distal 80% LAD lesion became apparent, and an overlapping 2.75 mm × 26 mm zotarolimus-eluting stent (Medtronic, Minneapolis, MN, USA) Resolute was advanced. During the delivery of the second stent, resistance was encountered at the proximal edge of the previously deployed stent. The primary mechanism of failure to cross was attributed to unfavorable strut interaction and strut snagging at the proximal stent margin. To overcome this, several supportive techniques were employed sequentially: initial attempts utilized deep guide catheter engagement to augment coaxial backup support, combined with high-torque extra-support guidewire (pilot 150) to alter wire bias. Unfortunately repeated manipulation resulted in entrapment, elongation, and severe deformation of the undeployed stent. Fluoroscopy demonstrated the stretched stent partially attached to the proximal LAD stent while freely mobile within the ascending aorta, creating a high risk of systemic embolization (Figure 2).

**Figure 3:**
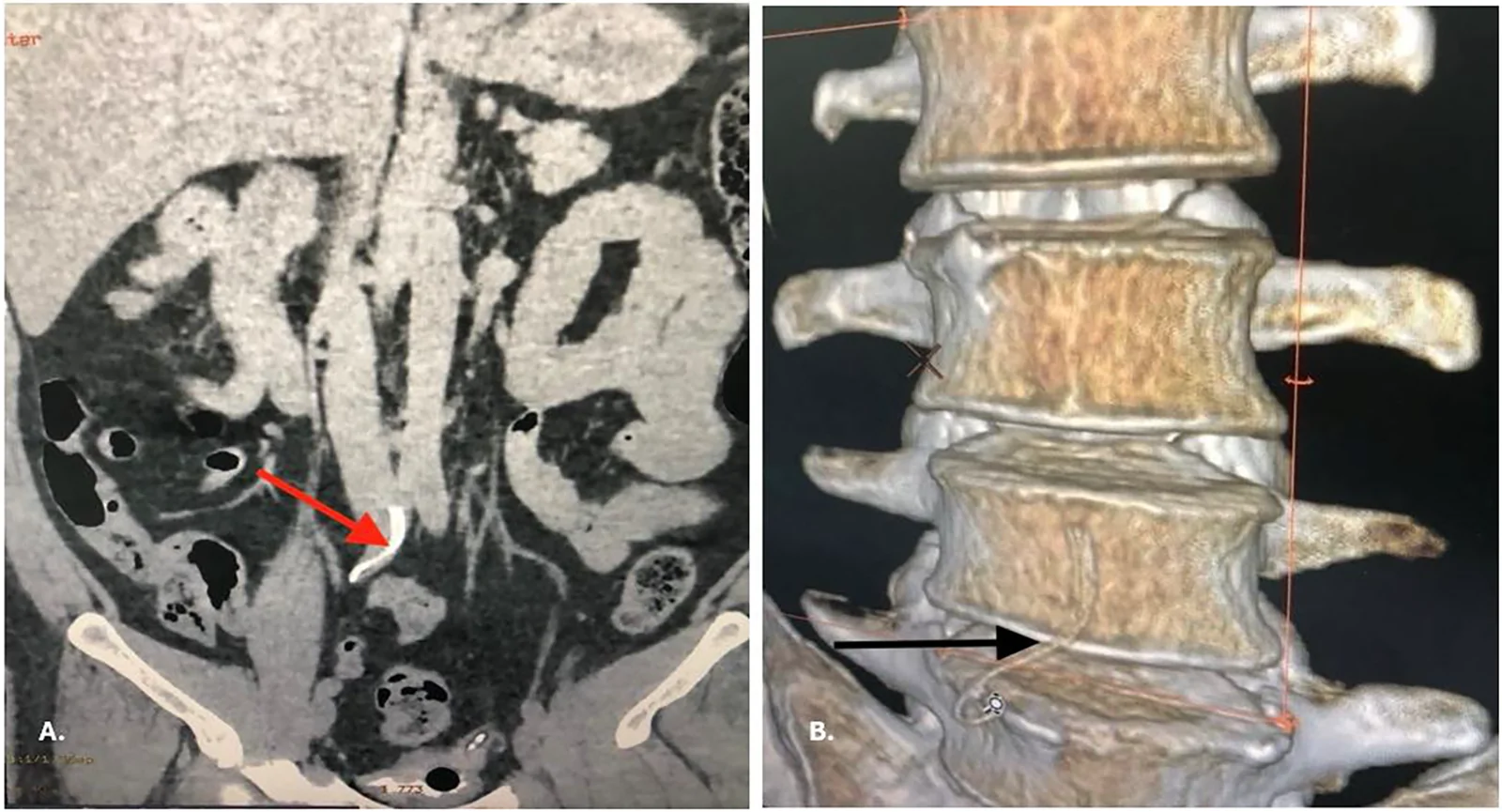
**(A)** Multiplanar coronal reformations. **(B)** Subsequent 3D volume-rendered reconstructions clearly delineating the radiopaque, metallic stent (red and black arrows) visualized lying along the expected trajectory of the right common iliac artery near the pelvic brim.

Following stent dislodgement, the patient experienced transient hemodynamic instability, characterized by hypotension with blood pressure dropping to 80/55 mmHg with sinus bradycardia and repeated vomiting. Immediate resuscitative measures were initiated in the cath lab: hemodynamic stability was promptly restored using continuous norepinephrine infusion titrated to maintain MAP >65 mmHg, IV Atropine 0.5 mg and fluid resuscitation with isotonic normal saline infusion. Once MAP and coronary perfusion were successfully stabilized, Percutaneous retrieval was performed using an 8F right femoral sheath for retrieval and an additional 7F left femoral sheath for backup access. A 10-mm Amplatz GooseNeck microsnare (Medtronic, Minneapolis, MN, USA) system was advanced through the right femoral sheath and successfully captured the hanging stent. During retrieval, however, traction on the deformed stent caused dislodgement of the previously implanted proximal LAD stent, which embolized distally and became impacted at the Right common iliac artery bifurcation. Localization of the second dislodged stent required immediate shifting the patient to CT for precise localization (Figures 3A,B).

The same snare system was subsequently used successfully to capture and retrieve the embolized iliac stent through the right femoral sheath without vascular complication.

**Figure 4:**
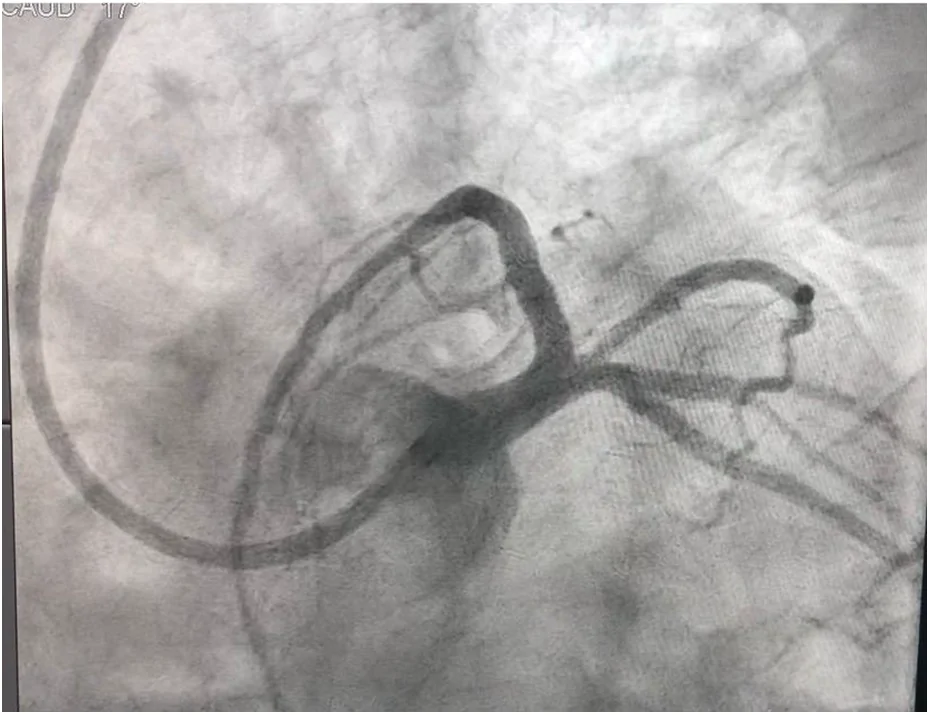
Left anterior oblique with caudal angulation angiographic view (spider) showing TIMI flow three (result of LAD angioplasty).

Following successful retrieval of both lost stents, the LAD was rewired using a PT2 MS guidewire, and definitive PCI was completed with implantation of (2.75 × 26 & 3.5 × 34) mm Resolute Integrity zotarolimus-eluting stents (Medtronic, Minneapolis, MN, USA) deployed at 16 atm with optimization of the overlap segment. Final angiography demonstrated an excellent procedural result with restoration of TIMI 3 flow and no residual stenosis (Figure 4).

The patient remained hemodynamically stable throughout the retrieval and revascularization procedures. Revascularization of the left anterior descending (LAD) artery was performed alongside successful management of the dislodged stents in one session. The procedure was briefly interrupted for an emergency whole body CT scan to achieve precise localization of the dislodged second stent, with the entire procedure completed within a 3-h timeframe.

The patient remained hemodynamically stable throughout hospitalization and was discharged after 3 days without any adverse events. During hospitalization, the patient was maintained on subcutaneous enoxaparin 60 mg twice daily in addition to dual antiplatelet therapy (DAPT), with DAPT prescribed for a one year following discharge. During follow-up, the patient remained asymptomatic with no recurrent cardiovascular or thromboembolic events over a 7-year follow-up period.

## Discussion

This case illustrates a rare and highly challenging complication of PCI involving sequential loss and embolization of two coronary stents. While historical rates were as high as 8%, contemporary incidences have fallen below 1% (estimated at 0.32%) due to improved low-profile, pre-mounted stent delivery systems (3, 6).

Common risk factors for dislodgement include severe vessel calcification, proximal tortuosity, and the presence of previously implanted stents (3, 4). In our case, the entrapment and subsequent deformation of the second stent likely occurred due to mechanical interference with the proximal edges of the first stent. This “stent-on-stent” interaction is a known mechanism for stent crumpling and failure to cross, which can lead to proximal migration or systemic embolization (4).

The most unique aspect of this case is the secondary dislodgement of a previously deployed stent during the retrieval of the first. While the Amplatz Goose Neck snare is a highly effective tool for capturing dislodged devices especially those that have moved off the guidewire, the traction force required to retrieve a deformed, stretched stent can create significant longitudinal stress (3).

In this scenario, the “stretched” configuration of the first stent likely acted as a hook, inadvertently snagging the proximal struts of the deployed LAD stent. This illustrates a rare but critical hazard of percutaneous retrieval: collateral dislodgement. Practitioners must remain vigilant for “floating” or “ring-like” configurations of lost stents in the aorta, as these can migrate rapidly to peripheral sites like the renal or iliac arteries (3, 6).

Kış et al. described a related complication in which a stent became dislodged from its balloon after entanglement with a previously implanted stent, and both stents were retrieved by snaring (7). Their report similarly demonstrates that resistance during retrieval may indicate attachment to another implanted stent. Our case differs in that the undeployed stent became markedly stretched and mobile in the ascending aorta, and traction during retrieval subsequently dislodged a successfully deployed proximal LAD stent, which embolized to the right common iliac artery bifurcation. This secondary aortic-to-iliac migration required urgent computed tomography for precise localization before the second snare maneuver.

It has been established that Percutaneous retrieval is now the standard of care, replacing surgical intervention in most cases (6). Several techniques exist as follows:

- Small-Balloon Technique: Most common when the guidewire position is maintained (6)
- Loop-Snare Technique: Preferred when the stent is “free-floating” or off the wire (3)
- Stent Crushing: A viable alternative when retrieval is impossible or too risky, involving the “crushing” of the lost stent against the vessel wall with a second stent (4, 6)

Percutaneous retrieval using a snare device remains the preferred approach for managing free or partially detached stents and can avoid the need for surgical intervention when performed successfully. Adequate guide support, large-bore access, and gentle catheter manipulation are important factors that improve retrieval success while minimizing the risk of additional embolization (3). Following successful retrieval, completion PCI was necessary to restore vessel patency and reduce the risk of acute thrombotic or ischemic complications. Overall, this case highlights the importance of recognizing stent loss early, using multimodal imaging when needed, and applying a careful stepwise interventional approach in the management of this rare but potentially serious complication. In our patient, the secondary embolization to the iliac bifurcation necessitated a second snare maneuver.

Data on the long-term consequences of stent loss and retrieval are scarce. However, successful retrieval without vascular injury typically leads to excellent outcomes (6). Our case is remarkable for its 7-year follow-up, demonstrating that even complex double-stent complications can be managed without late-term thromboembolic or cardiovascular sequelae, provided that definitive revascularization is optimized following retrieval.

## Conclusion

Double coronary stent loss with sequential embolization is an exceptionally rare and high-risk complication of PCI. This case demonstrates that even complex scenarios involving aortic migration and peripheral embolization can be successfully managed without surgery. Early recognition, appropriate imaging, and operator expertise are key determinants of success.

## Learning points

- Double stent loss is extremely rare but potentially catastrophic
- Retrieval attempts may cause secondary embolization
- Snare retrieval is effective even in complex multi-site embolization
- Percutaneous management can avoid surgery in experienced hands

## Data Availability

The original contributions presented in the study are included in the article/Supplementary Material, further inquiries can be directed to the corresponding author.
